# Supplementary figures and images for: Hypoxic bone marrow mesenchymal stem cell exosomes promote angiogenesis and enhance endometrial injury repair through the miR-424-5p-mediated DLL4/Notch signaling pathway
Source: PeerJ. 2024 Feb 22;12:e16953. doi: 10.7717/peerj.16953 (PMC10894593; doi:10.7717/peerj.16953)

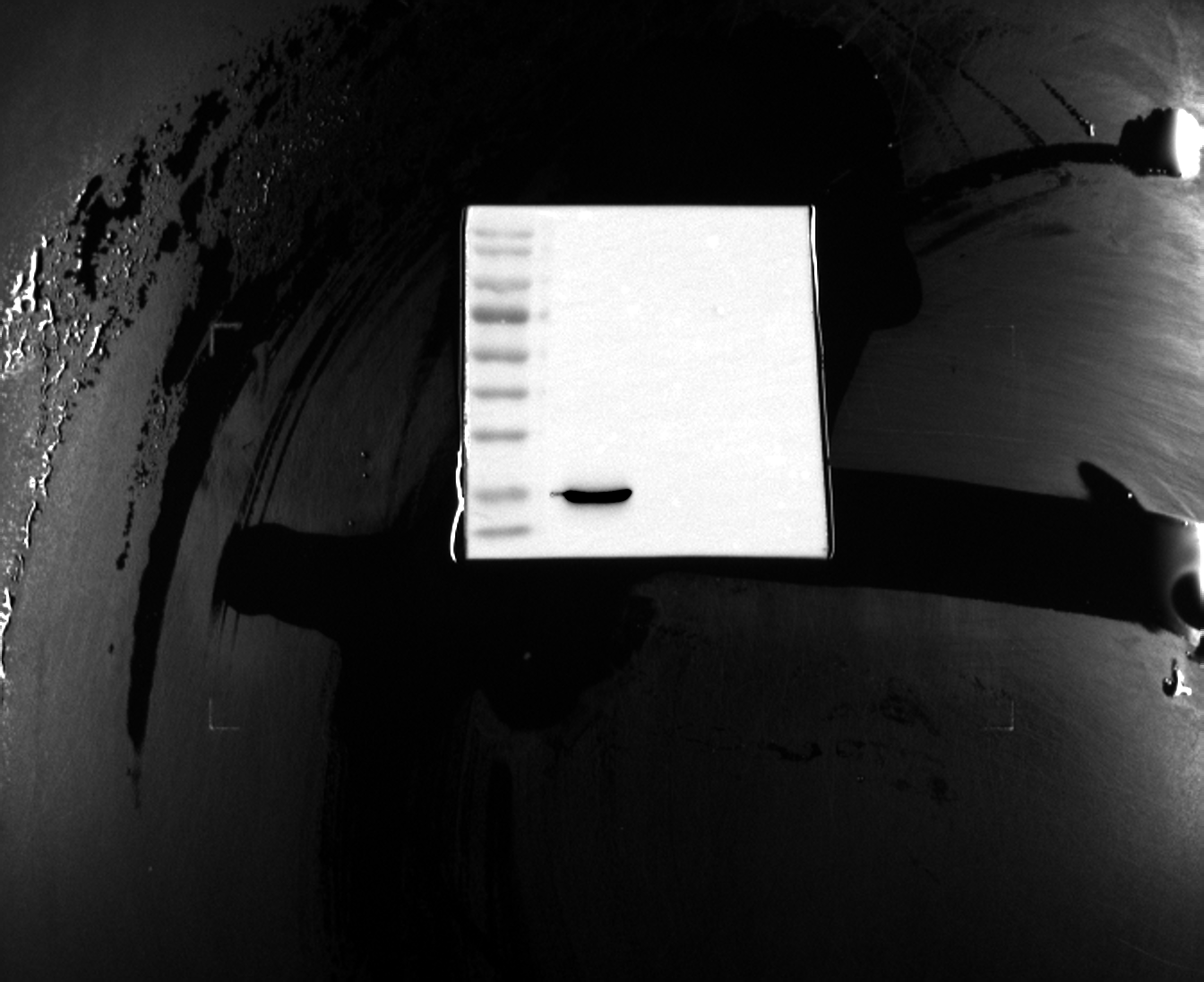

Supplement: Supplemental Information 2 [file peerj-12-16953-s002.zip › Figure 1C/1-CD9.tif]

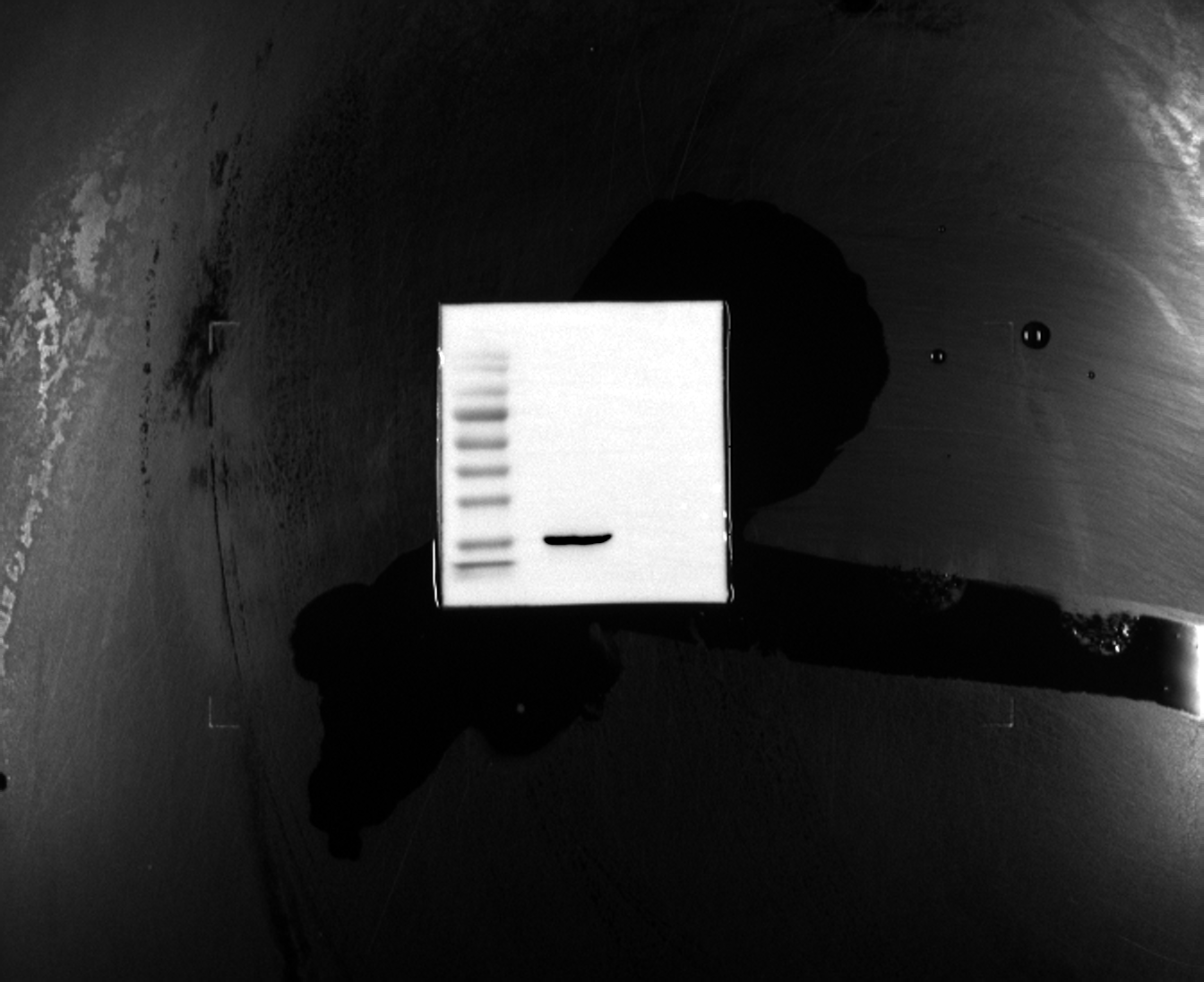

Supplement: Supplemental Information 2 [file peerj-12-16953-s002.zip › Figure 1C/2-CD63.tif]

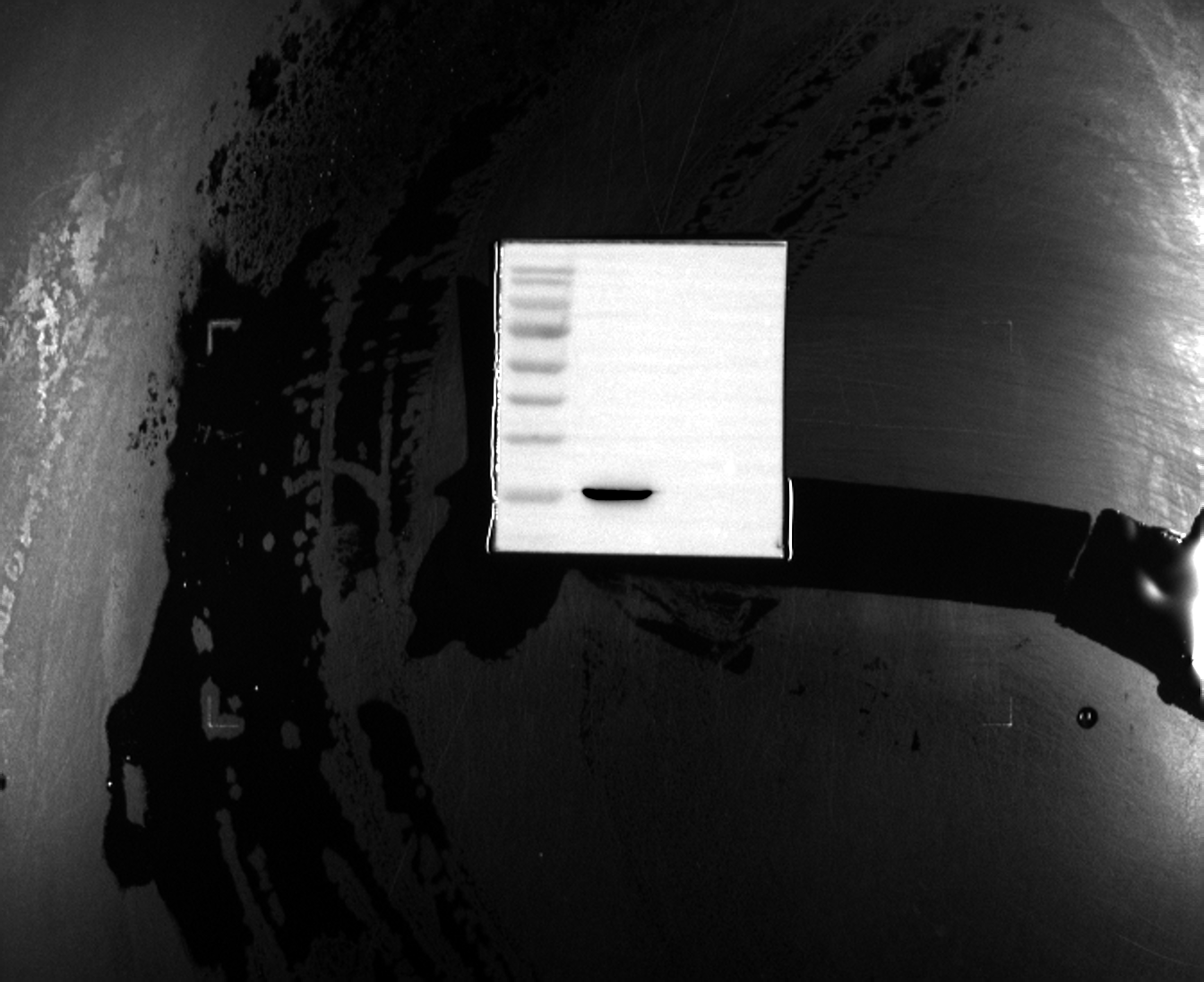

Supplement: Supplemental Information 2 [file peerj-12-16953-s002.zip › Figure 1C/3-CD81.tif]

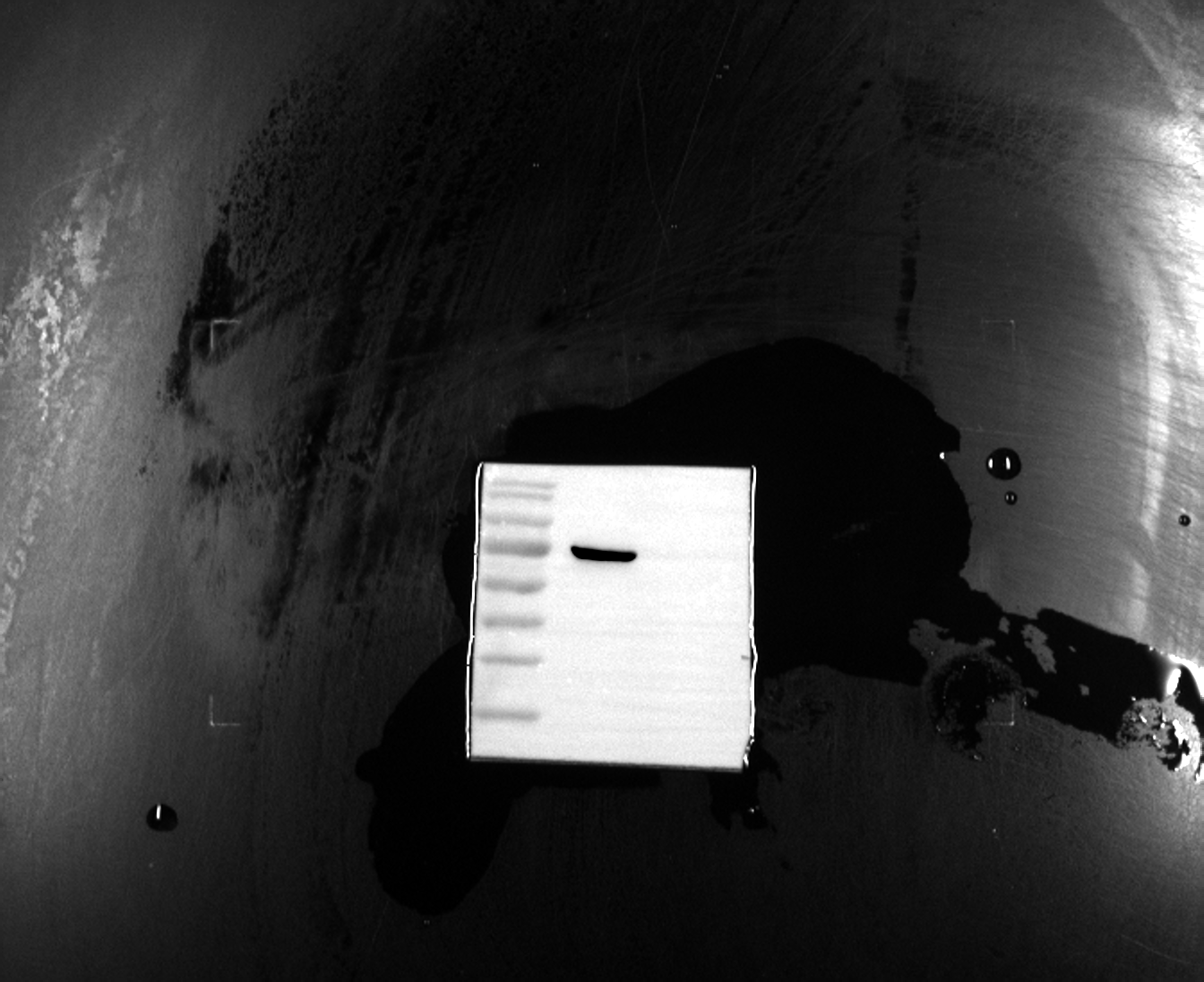

Supplement: Supplemental Information 2 [file peerj-12-16953-s002.zip › Figure 1C/4-HSP70.tif]

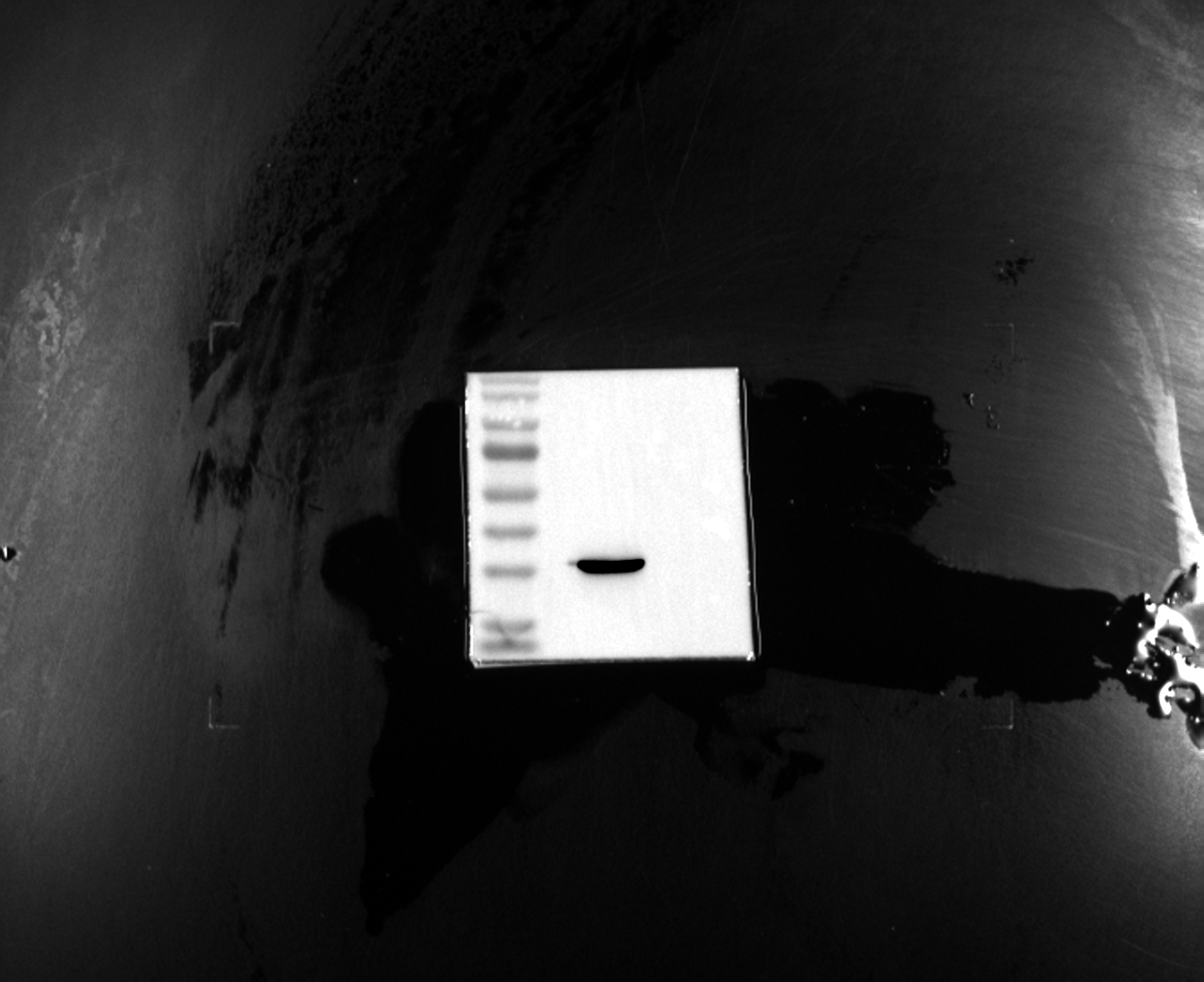

Supplement: Supplemental Information 2 [file peerj-12-16953-s002.zip › Figure 1C/5-GAPDH.tif]

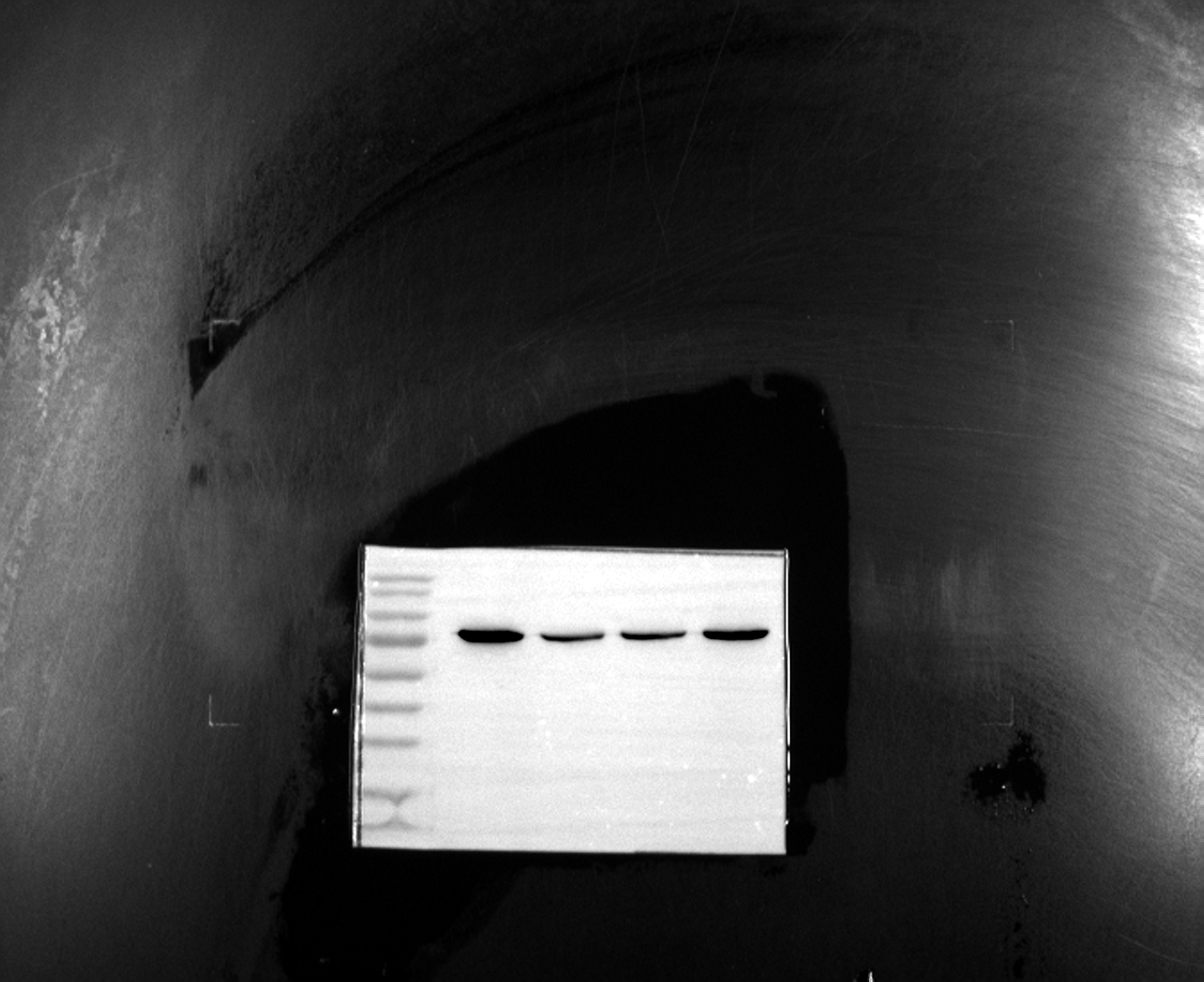

Supplement: Supplemental Information 2 [file peerj-12-16953-s002.zip › Figure 3A/1-DLL4.tif]

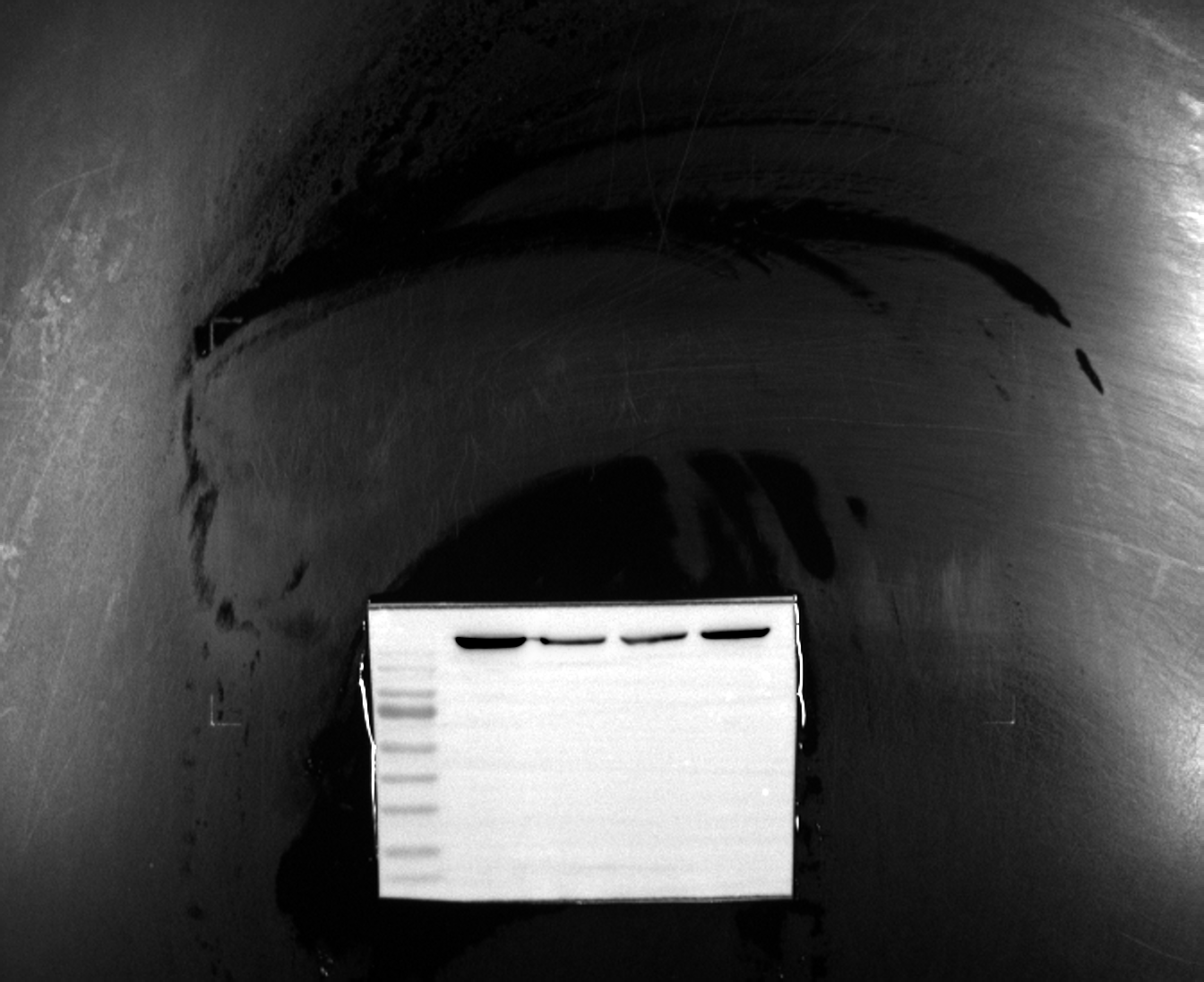

Supplement: Supplemental Information 2 [file peerj-12-16953-s002.zip › Figure 3A/2-Noth1.tif]

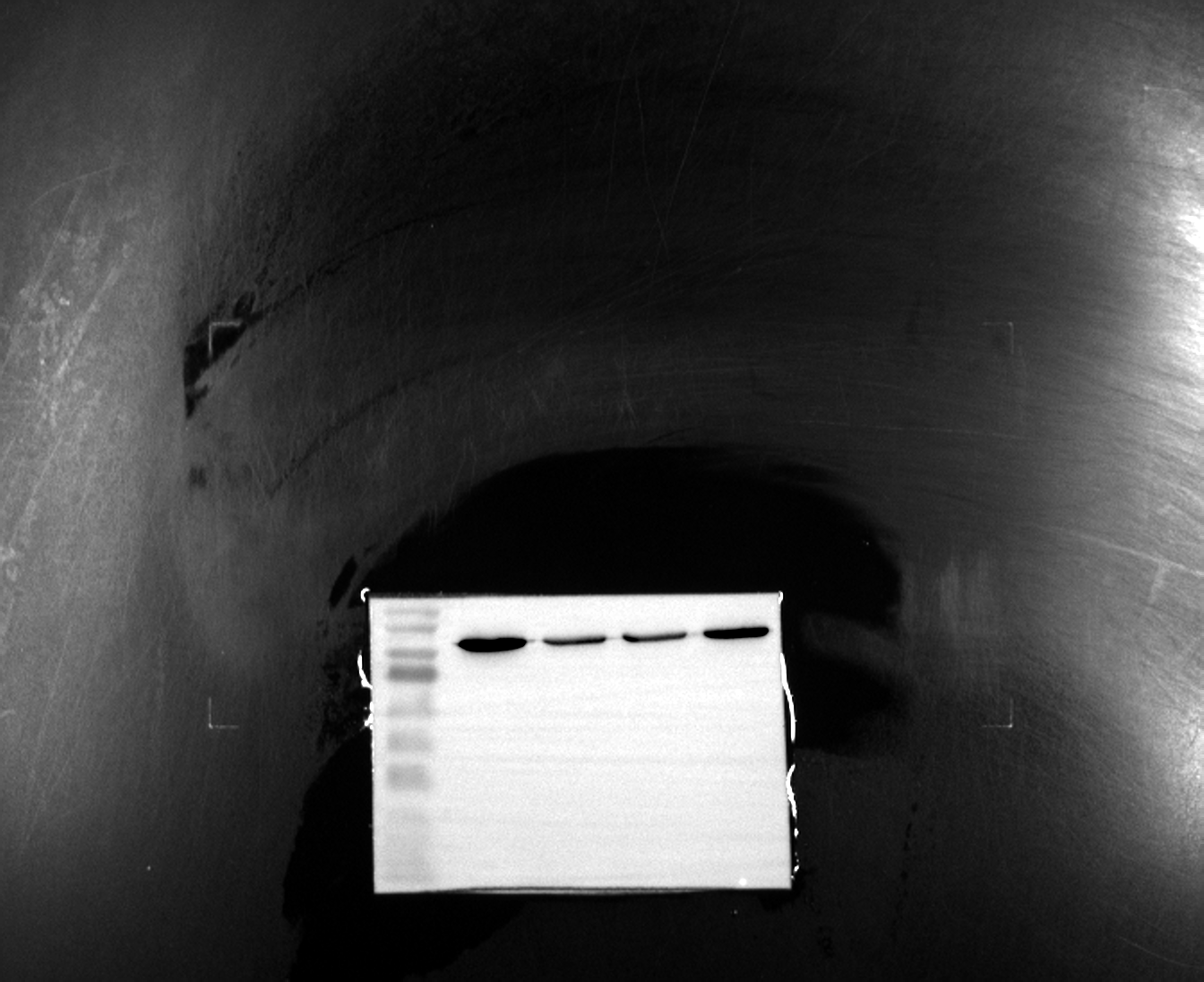

Supplement: Supplemental Information 2 [file peerj-12-16953-s002.zip › Figure 3A/3-Noth2.tif]

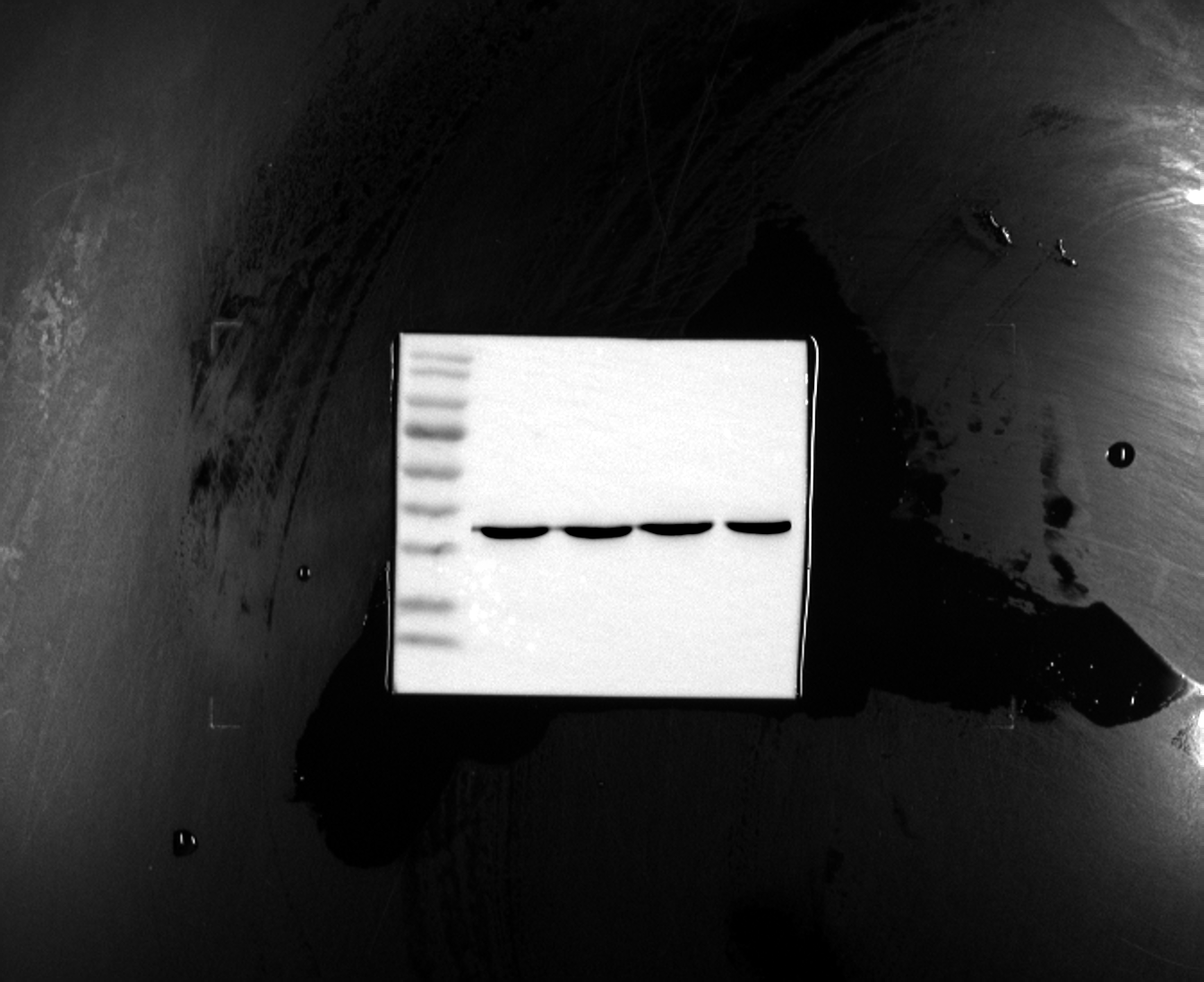

Supplement: Supplemental Information 2 [file peerj-12-16953-s002.zip › Figure 3A/4-GAPDH.tif]

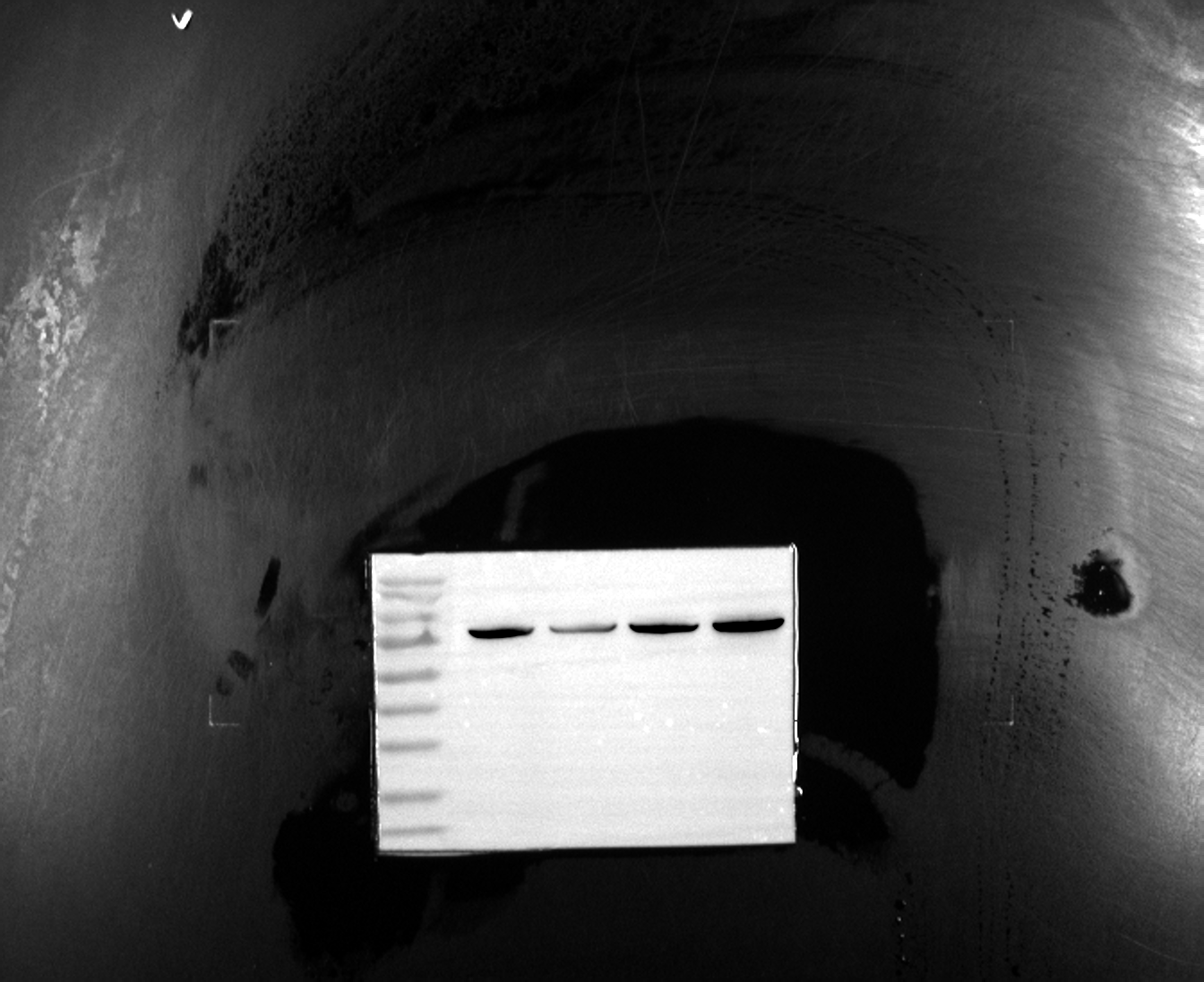

Supplement: Supplemental Information 2 [file peerj-12-16953-s002.zip › Figure 4C/1-DLL4.tif]

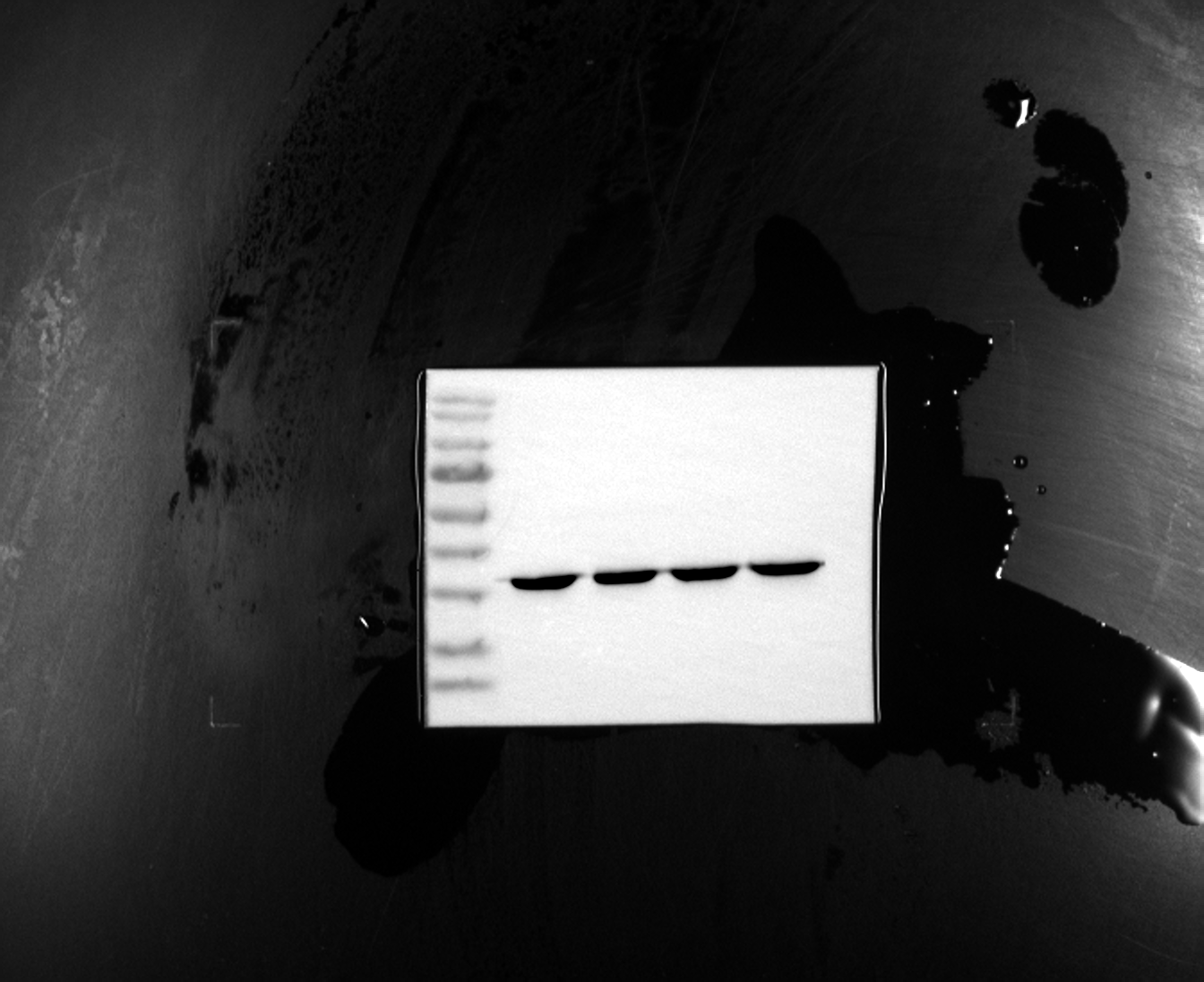

Supplement: Supplemental Information 2 [file peerj-12-16953-s002.zip › Figure 4C/2-GAPDH.tif]

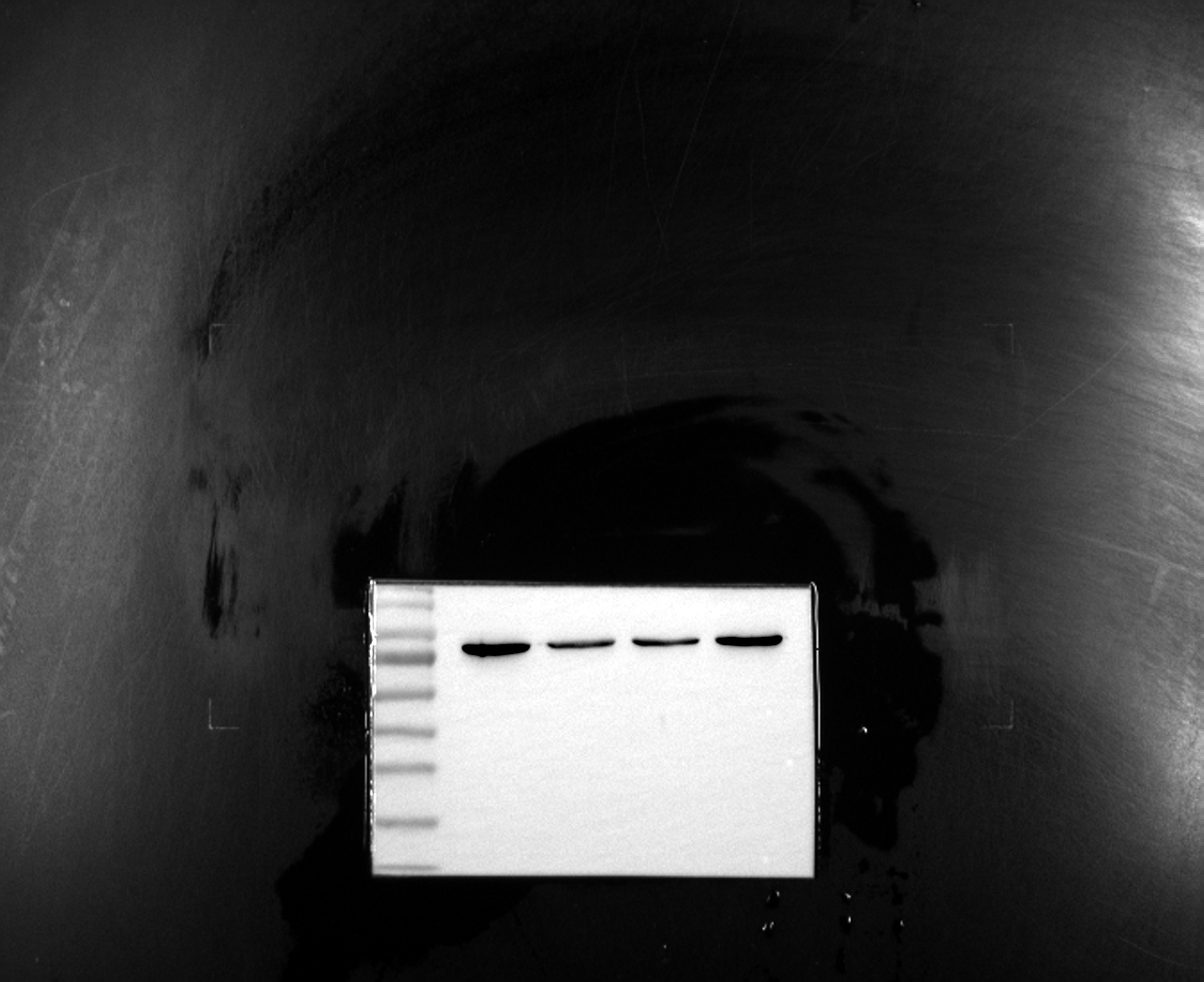

Supplement: Supplemental Information 2 [file peerj-12-16953-s002.zip › Figure 4D/1-DLL4.tif]

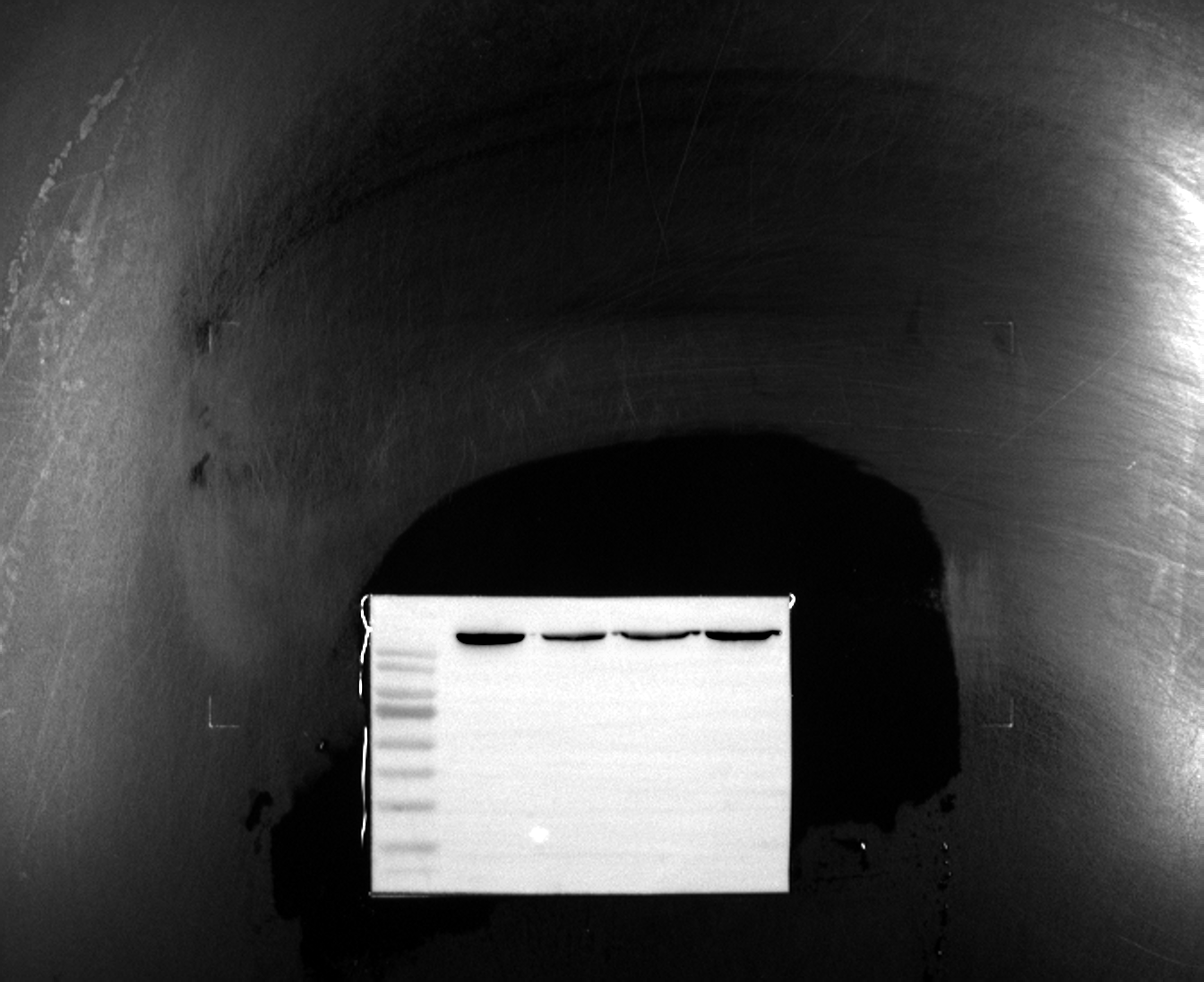

Supplement: Supplemental Information 2 [file peerj-12-16953-s002.zip › Figure 4D/2-Noth1.tif]

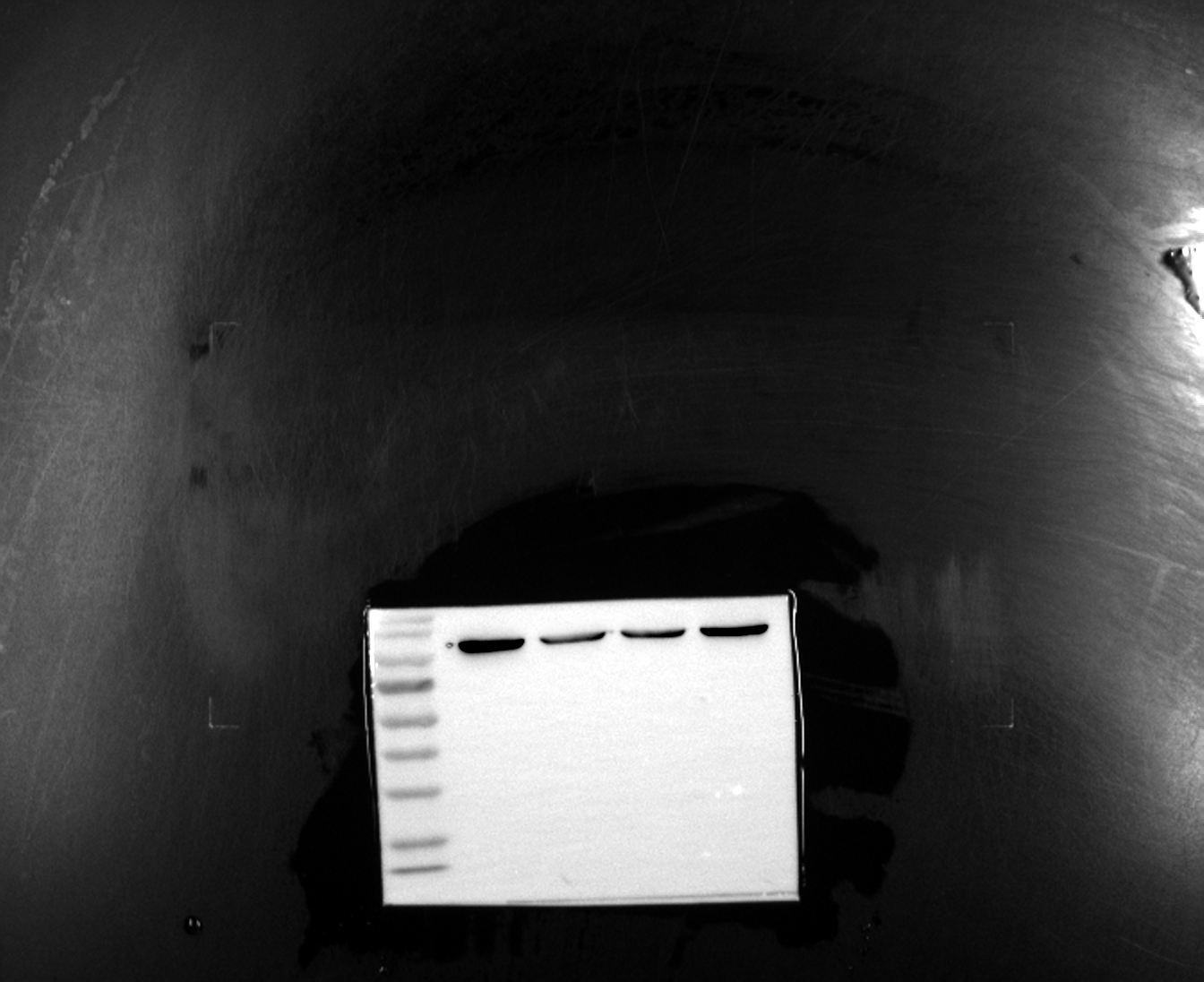

Supplement: Supplemental Information 2 [file peerj-12-16953-s002.zip › Figure 4D/3-Noth2.tif]

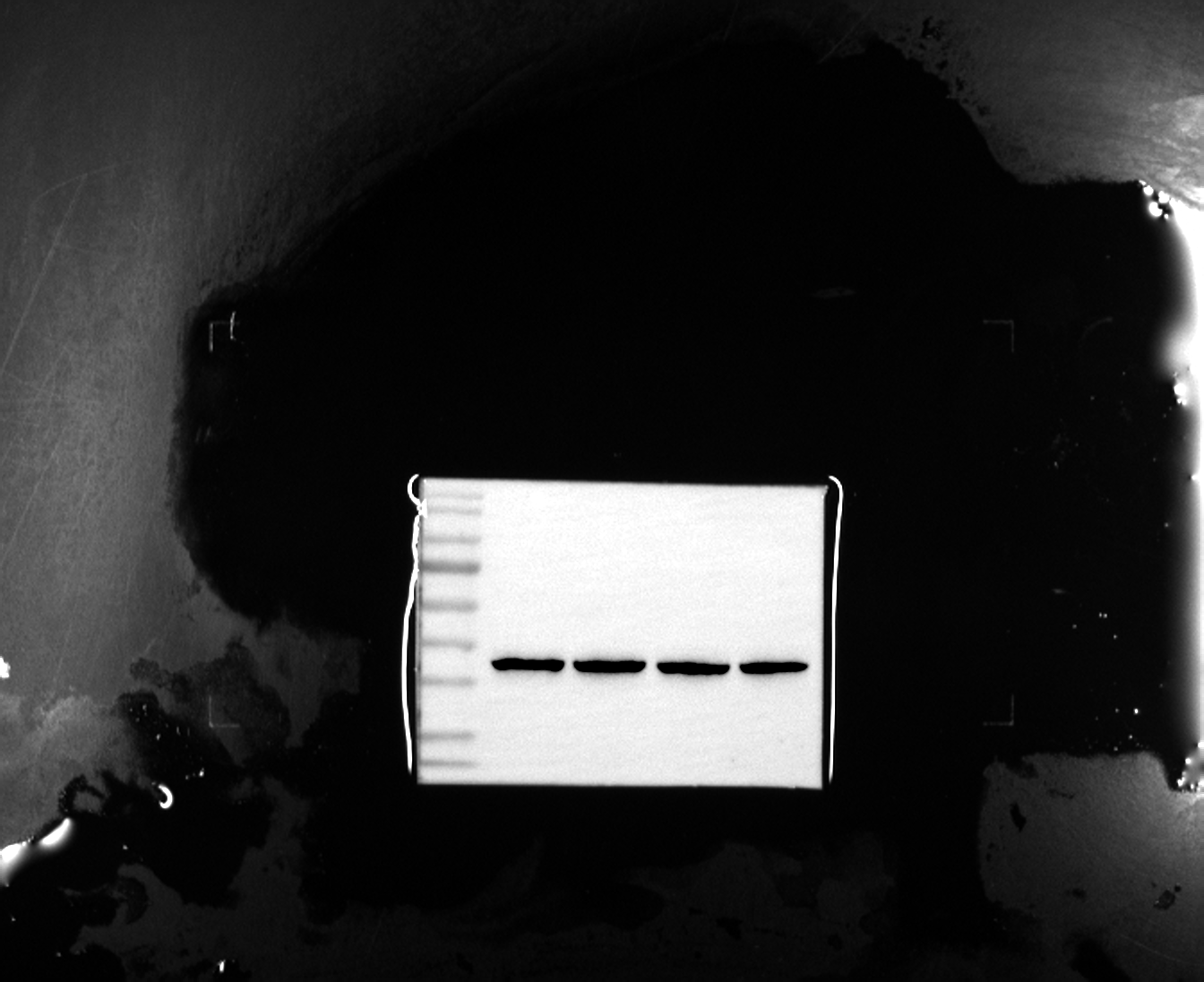

Supplement: Supplemental Information 2 [file peerj-12-16953-s002.zip › Figure 4D/4-GAPDH.tif]

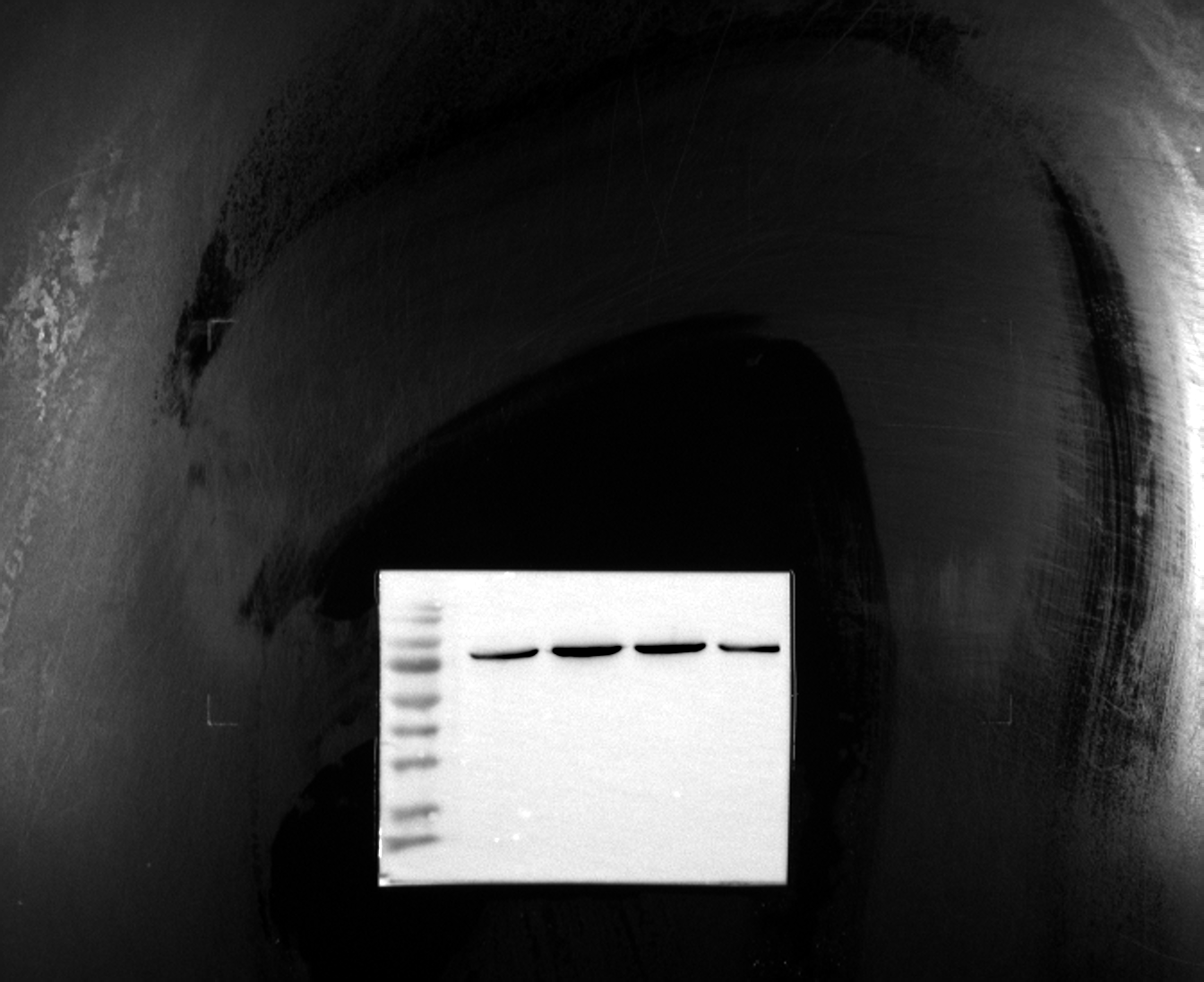

Supplement: Supplemental Information 2 [file peerj-12-16953-s002.zip › Figure 6D/1-DLL4.tif]

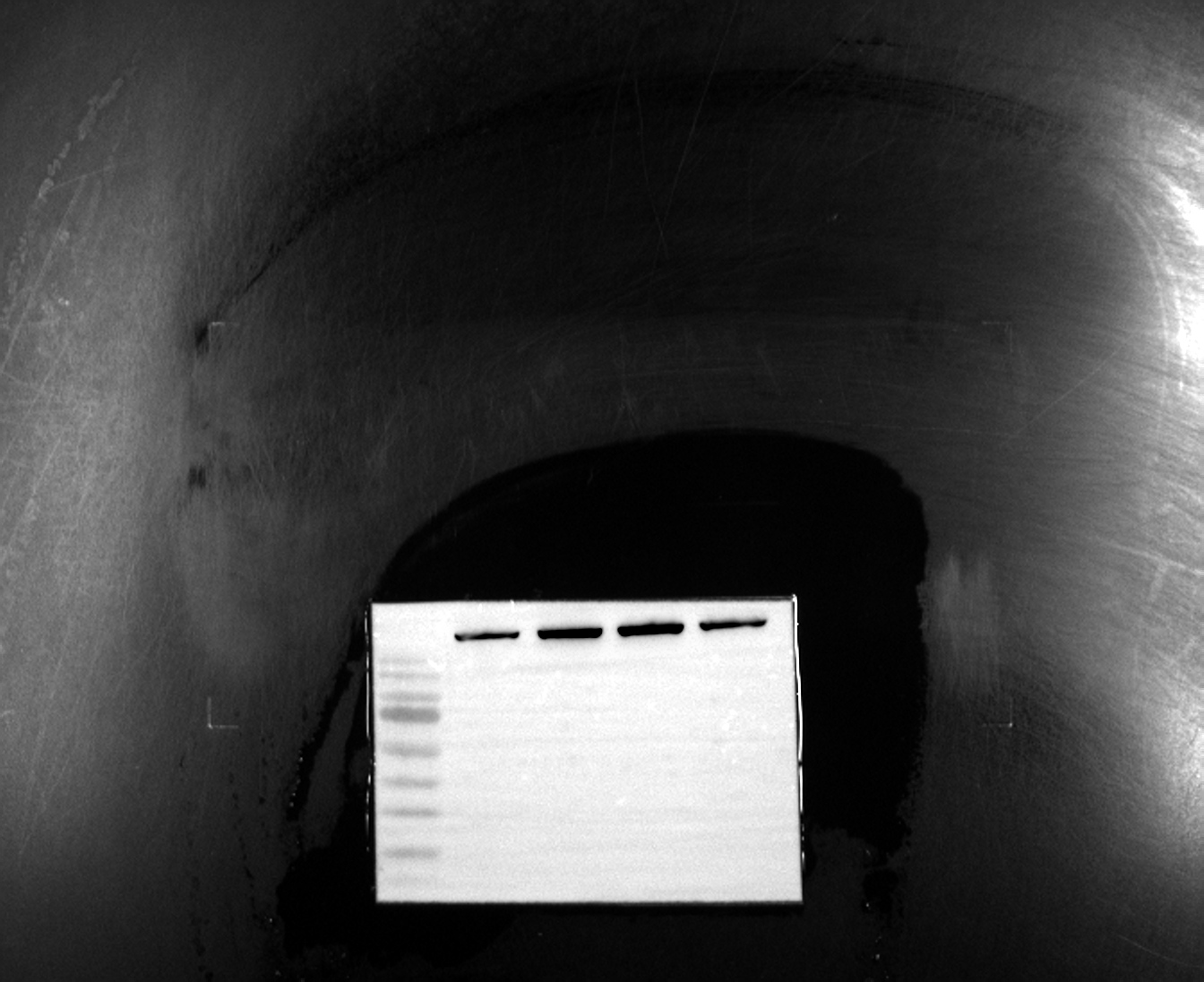

Supplement: Supplemental Information 2 [file peerj-12-16953-s002.zip › Figure 6D/2-Noth1.tif]

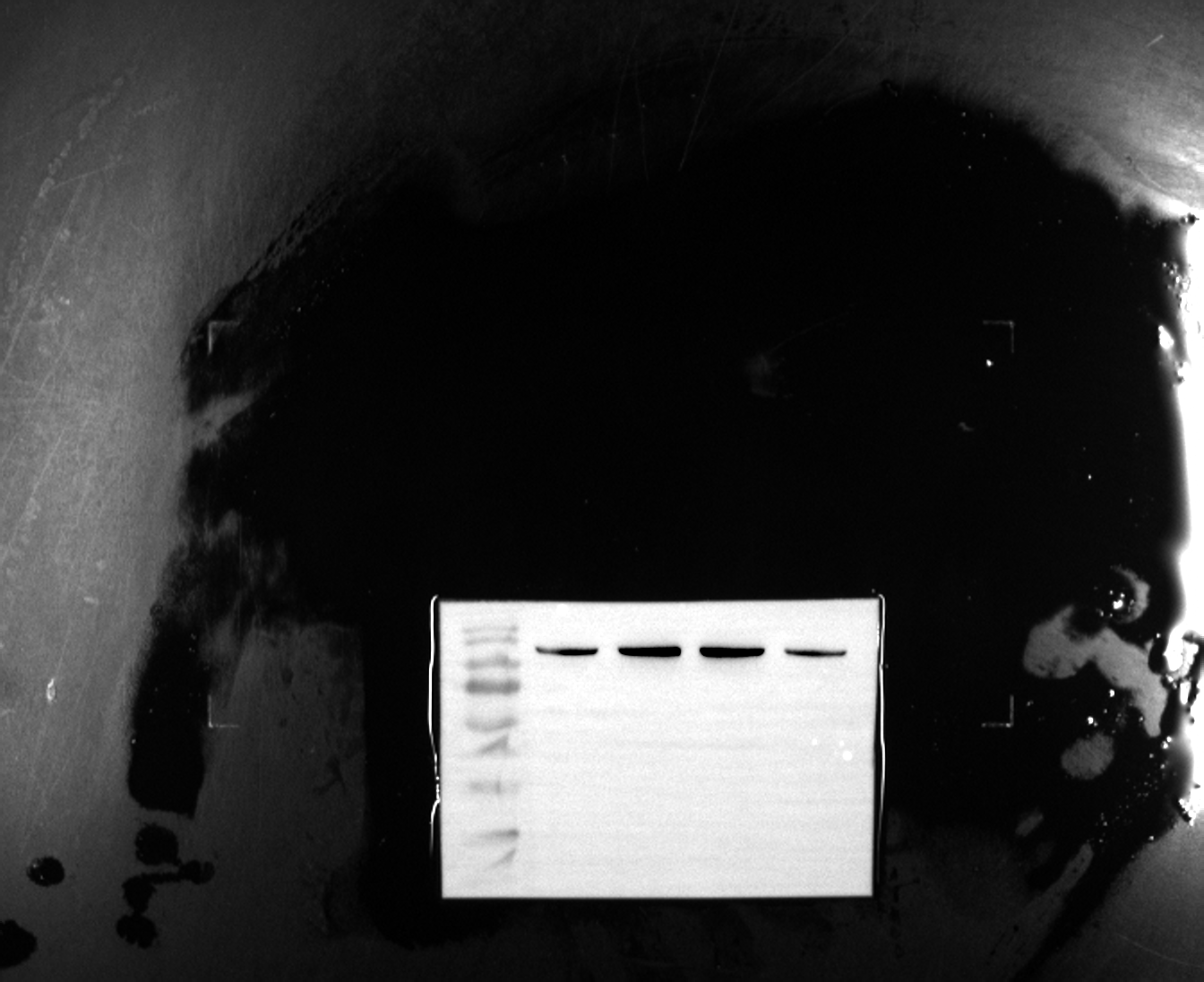

Supplement: Supplemental Information 2 [file peerj-12-16953-s002.zip › Figure 6D/3-Noth2.tif]

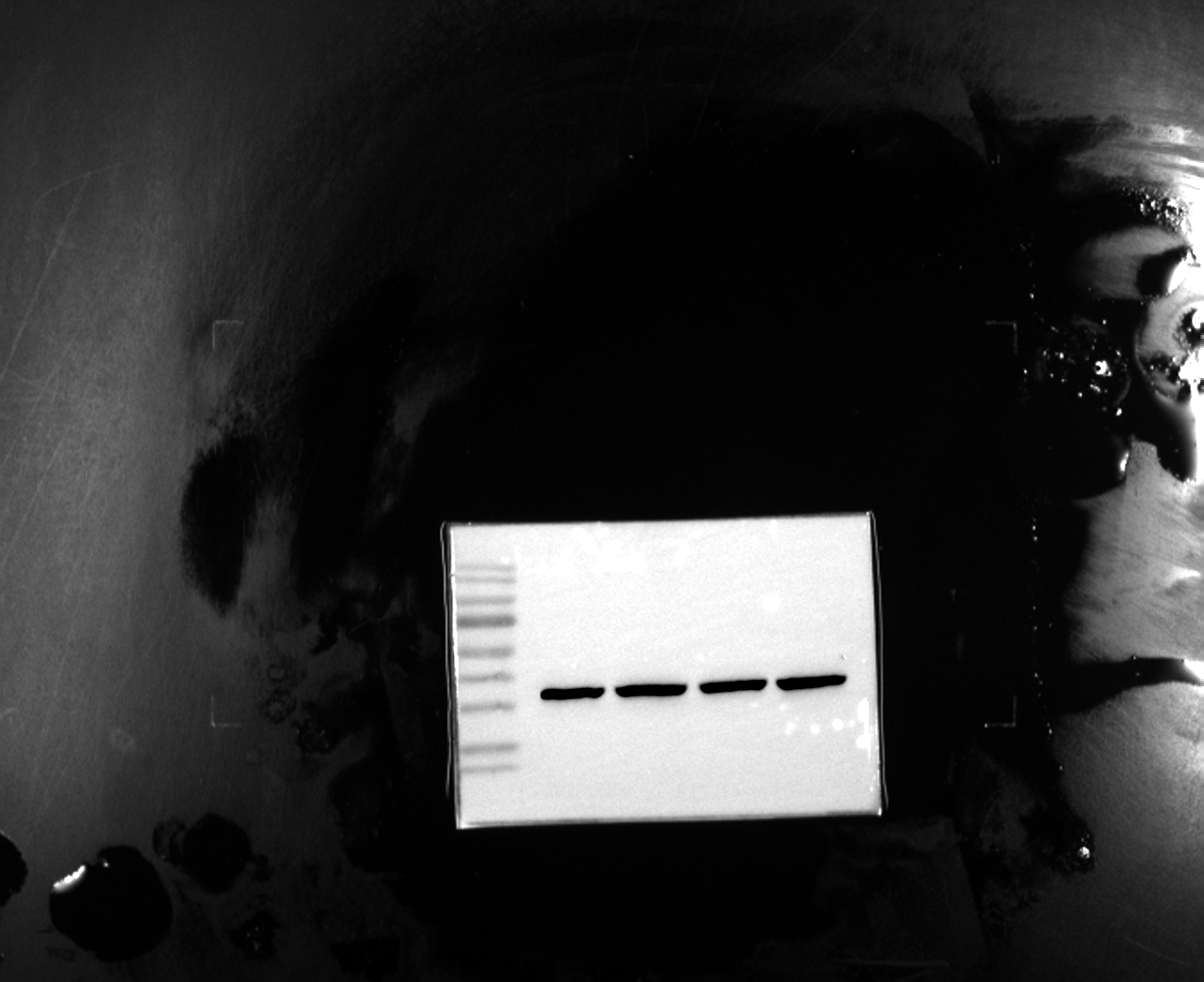

Supplement: Supplemental Information 2 [file peerj-12-16953-s002.zip › Figure 6D/4-GAPDH.tif]

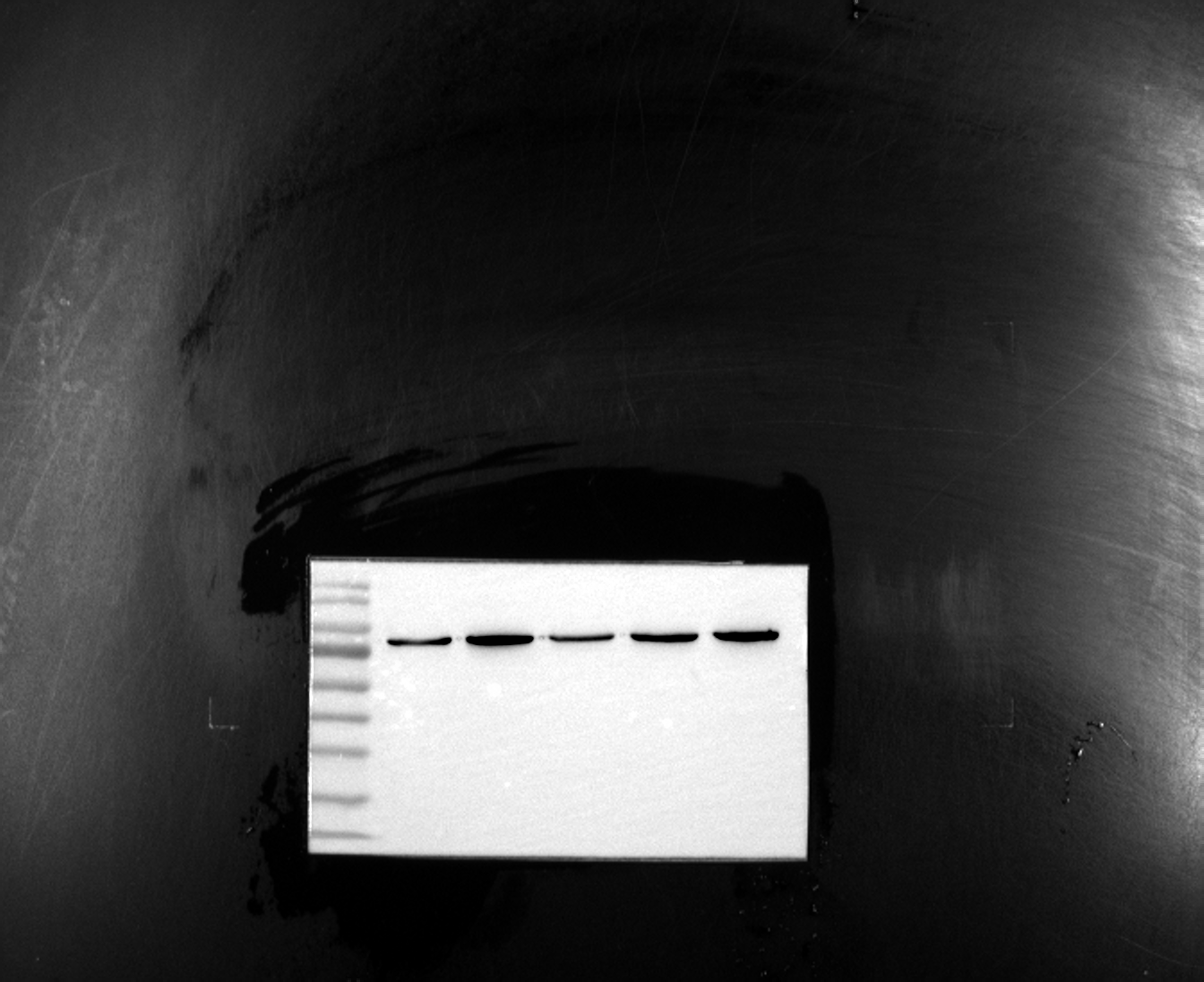

Supplement: Supplemental Information 2 [file peerj-12-16953-s002.zip › Figure 7D/1-DLL4.tif]

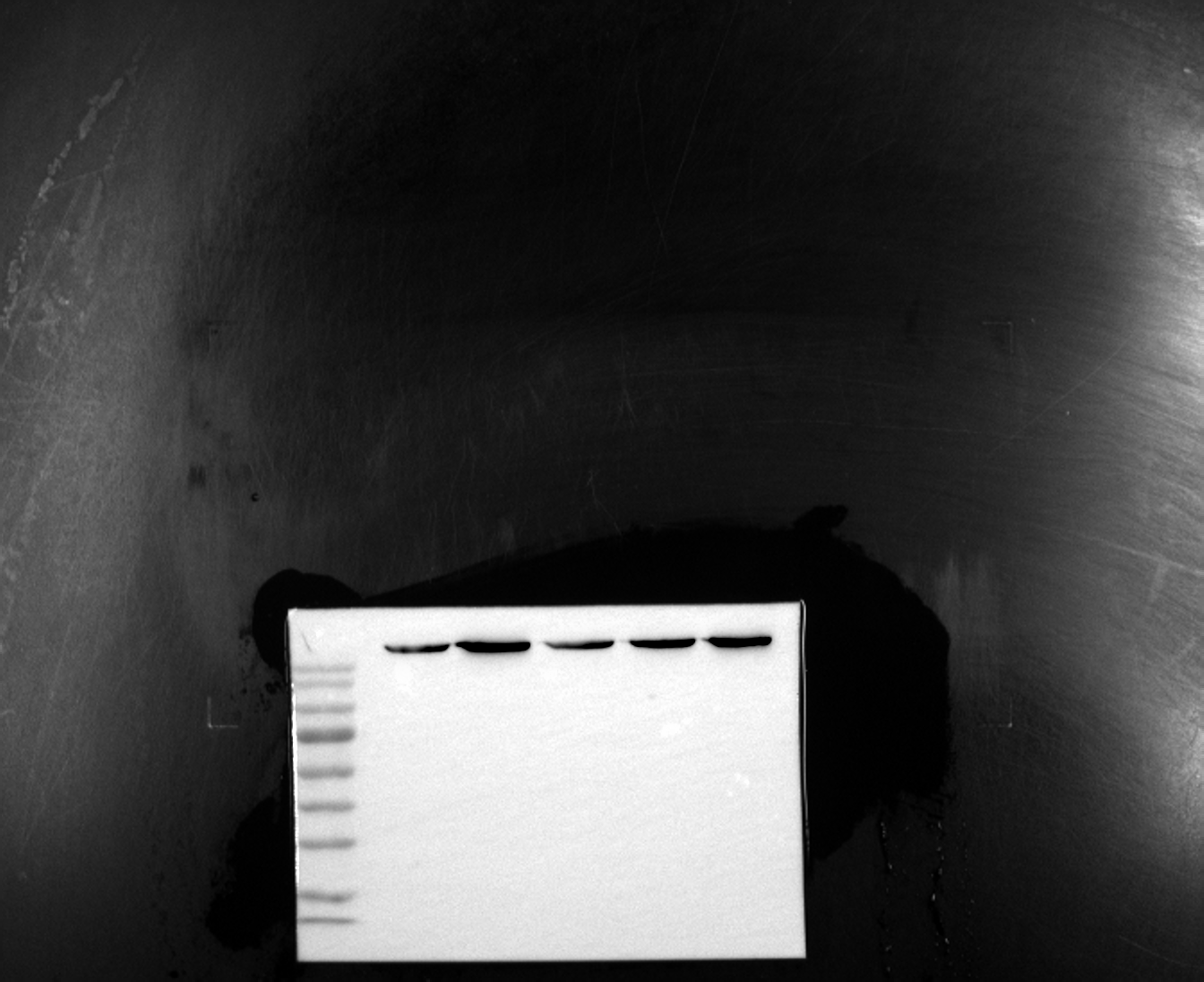

Supplement: Supplemental Information 2 [file peerj-12-16953-s002.zip › Figure 7D/2-Noth1.tif]

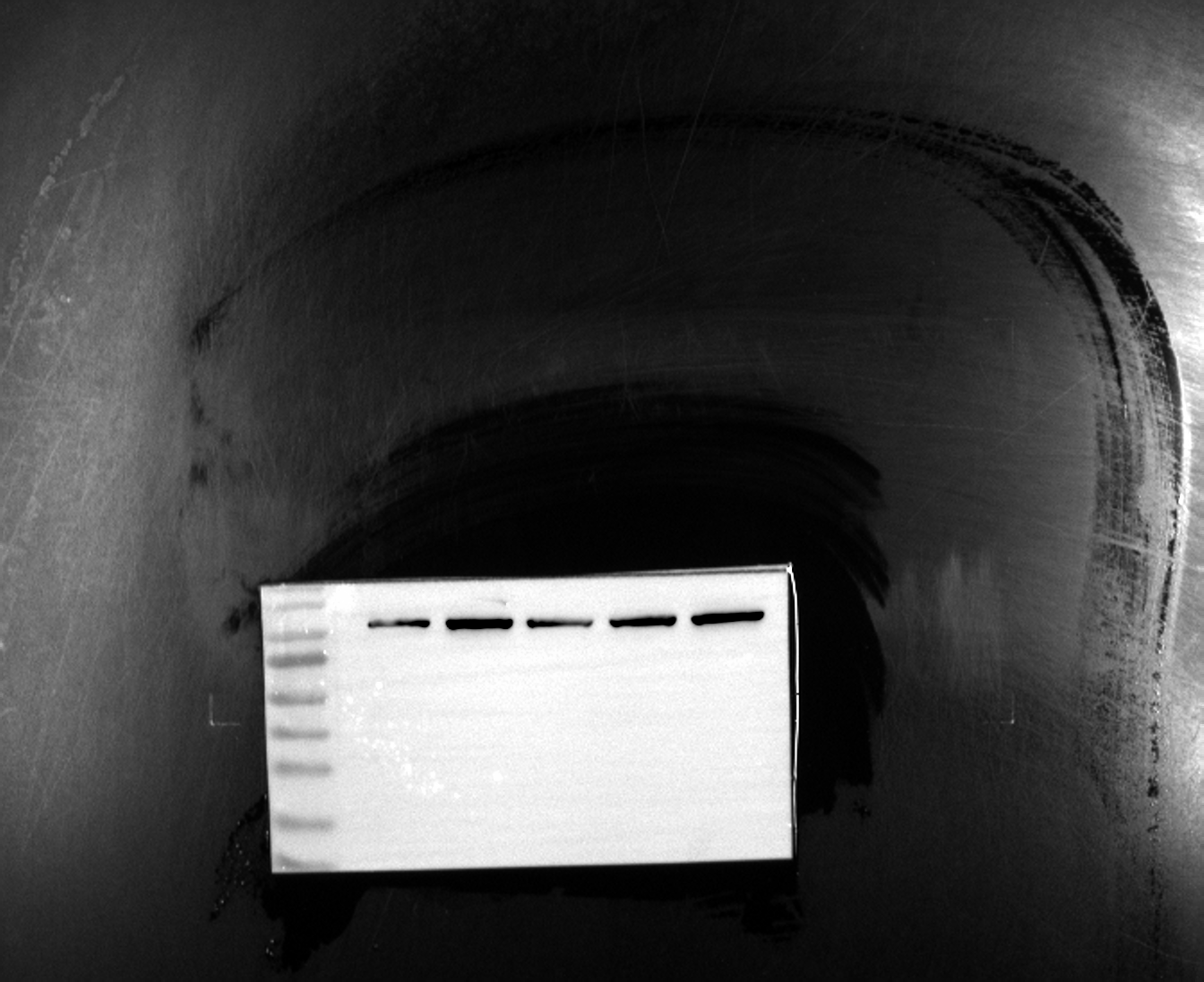

Supplement: Supplemental Information 2 [file peerj-12-16953-s002.zip › Figure 7D/3-Noth2.tif]

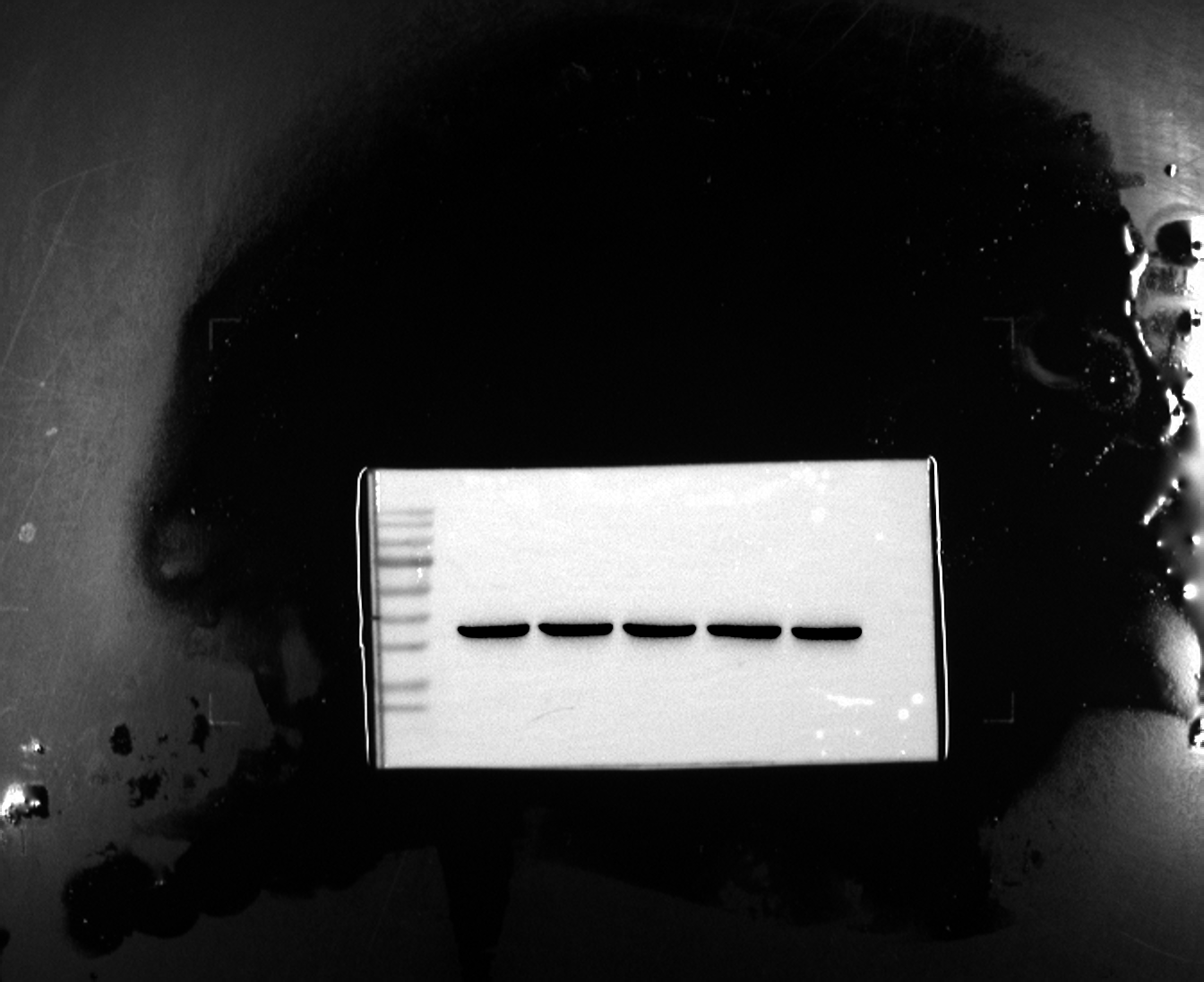

Supplement: Supplemental Information 2 [file peerj-12-16953-s002.zip › Figure 7D/4-GAPDH.tif]
